# GICP-Based Registration Flow Improvement and Planar Consistency Evaluation for Heterogeneous Multi-LiDAR Systems in Grain Warehousing Robots

**DOI:** 10.3390/s26113447

**Published:** 2026-05-29

**Authors:** Lan Wu, Haozhe Wang, Qian Li

**Affiliations:** 1 School of Mechanical and Electrical Engineering, Henan University of Technology, Zhengzhou 450001, China; whz8848thj@163.com; 2 COFCO Engineering & Technology (Zhengzhou) Co., Ltd., Zhengzhou 450001, China; 18638136860@163.com

**Keywords:** heterogeneous multi-LiDAR, point cloud registration, GICP, grain warehousing robot, low-overlap point clouds, planar consistency evaluation

## Abstract

**Highlights:**

**What are the main findings?**

- A GICP-based registration flow improvement method was developed for heterogeneous multi-LiDAR systems in grain warehousing robots, combining overlap-region cropping, voxel downsampling, and a star-topology registration strategy.
- The proposed method achieved lower point-to-plane errors than ICP, point-to-plane ICP, and NDT in both sparse–dense and sparse–sparse registration tasks, while maintaining acceptable computational efficiency.

**What are the implications of the main findings?**

- The results show that selecting a reference LiDAR according to overlap relationships, rather than only point density, can improve the stability of heterogeneous multi-LiDAR registration.
- The introduced planar consistency evaluation provides a practical way to assess structural alignment quality, which is valuable for perception, mapping, and autonomous operation in grain warehousing robots.

**Abstract:**

Grain intake is a key operation in grain storage that directly affects storage efficiency, operational safety, and grain quality. In grain-entry scenarios, single LiDAR sensors are easily limited by blind spots and occlusions, making multi-LiDAR collaborative perception necessary for reliable three-dimensional environment sensing. However, heterogeneous LiDARs differ in scan lines, point density, viewing angle, installation pose, and noise characteristics, which leads to low-overlap and mixed sparse–dense point cloud registration challenges. To address this issue, this paper proposes a GICP-based registration flow improvement method for heterogeneous multi-LiDAR systems used in intelligent grain warehousing robots. The method improves registration stability through overlap-region cropping, voxel downsampling, and a star-topology registration strategy, and further introduces a point-to-plane evaluation metric based on local planar models together with cross-LiDAR planar consistency verification. Experimental results show that the proposed method reduces the point-to-plane error to 0.1487 m in the $L_{0}-L_{1}$ registration task and 0.1090 m in the $L_{1}-L_{2}$ registration task, outperforming ICP, point-to-plane ICP, and NDT while maintaining acceptable computational efficiency. These results demonstrate that the method can improve structural alignment quality and provide reliable geometric support for multi-sensor perception, mapping, and autonomous operation of grain warehousing robots. Rather than proposing a fundamentally new registration mathematical model, this study proposes a highly engineered GICP-based workflow. It should be noted that the proposed workflow is specifically tailored and optimized for plane-dominated and semi-static grain storage environments, restricting its validated scope to static or low-speed multi-LiDAR registration tasks.

## 1. Introduction

### 1.1. Research Background

Grain storage is a fundamental component of food security, and grain intake is a key operation that directly influences storage efficiency, operational safety, and grain quality. At present, grain intake in many storage facilities still relies on manual labor or semi-automated equipment, which is often associated with high dust concentration, enclosed working spaces, heavy labor intensity, and considerable safety risks. With the development of smart granaries and unmanned warehousing, higher requirements have been imposed on the automation, intelligence, and continuous operation capability of grain-entry equipment. Therefore, it is of practical significance to develop intelligent grain warehousing robots with autonomous perception, environment modeling, and accurate localization capabilities.

LiDAR has become an important sensing modality for mobile robots because of its strong resistance to illumination changes, high ranging accuracy, and suitability for complex industrial environments. However, in grain-entry scenarios, the workspace is usually large, structurally complex, and frequently affected by occlusions caused by equipment and facilities. A single LiDAR sensor is often insufficient to simultaneously provide reliable far-range mapping and near-range blind-zone coverage. For this reason, multi-LiDAR collaborative perception has become an effective solution for improving environmental coverage and sensing robustness. Nevertheless, LiDAR sensors of different types usually differ in scan lines, point density, field of view, installation pose, and noise characteristics, which leads to heterogeneous point clouds and makes cross-sensor registration significantly more challenging.

To address these issues, this study focuses on low-overlap point cloud registration for heterogeneous multi-LiDAR systems mounted on grain warehousing robots. From the perspective of engineering deployment, the study investigates registration workflow design, overlap-region constraints, and result evaluation strategies, thereby improving the stability of point cloud fusion and the alignment quality of structural surfaces. In addition, a point-to-plane metric based on local planar models and a cross-LiDAR planar consistency verification scheme is introduced to better characterize the geometric consistency of the registration results.

### 1.2. Related Work

Point cloud registration is a key problem in three-dimensional environment perception, map construction, and multi-sensor fusion. Its core task is to estimate the optimal rigid transformation between point clouds represented in different coordinate systems. Existing studies can be broadly divided into homogeneous point cloud registration and heterogeneous point cloud registration according to sensor type and data source. Among homogeneous registration methods, the iterative closest point (ICP) algorithm is one of the most classical approaches [1]. ICP repeatedly establishes correspondences and minimizes point-to-point distances. Although it is simple and effective, it is sensitive to the initial pose and may converge to local optima in the presence of noise, repeated structures, or low overlap. To improve registration in planar environments, point-to-plane ICP was proposed by defining the residual as the distance from a source point to the local plane of the target point cloud, thereby improving both accuracy and convergence speed in large planar scenes [2].

On this basis, generalized iterative closest point (GICP) introduces local neighborhood geometry into the registration process and uses covariance matrices to probabilistically model local shape characteristics. As a result, point-to-point and point-to-plane constraints can be handled in a unified framework. Compared with standard ICP, GICP generally exhibits better robustness and geometric adaptability, and it has therefore been widely used in LiDAR mapping, localization, and multi-sensor calibration [3]. However, when point clouds are acquired from different types of LiDAR sensors, differences in scan lines, point density, noise distribution, and sampling mode may still lead to unstable correspondences, limited overlap, and inconsistent local structure representation. Consequently, the engineering performance of standard GICP still leaves room for improvement in sparse–dense mixed and low-overlap scenarios.

For heterogeneous point cloud registration, existing studies mainly focus on feature matching, probabilistic modeling, and learning-based methods. Planar objects have also been used for extrinsic calibration of multiple 3D LiDAR sensors, demonstrating the effectiveness of planar constraints in improving geometric alignment [4]. Multi-LiCa further adopts a motion- and targetless two-step pipeline with feature-based coarse alignment and GICP-based fine registration for partially overlapping multi-LiDAR systems [5]. In addition, GMMCalib formulates LiDAR extrinsic calibration as a GMM-based joint registration problem and improves robustness against local minima and initialization sensitivity [6]. For narrow-field-of-view multi-LiDAR systems, synergistic planar and circular features have also been used to improve calibration accuracy [7]. Natural-environment-based self-calibration has likewise been investigated to reduce reliance on dedicated calibration targets in practical deployments [8]. Recent work has further addressed low-overlap multi-source LiDAR registration through dual-stage feature pruning and progressive hierarchical strategies [9], as well as displacement-corrected geometric consistency to enhance robust 3D sensing under limited overlapping conditions [10]. Moreover, improved ICP-based variants continue to be explored for enhancing robustness and alignment accuracy in point cloud registration [11]. Beyond these recent multi-LiDAR calibration and registration studies, some methods establish cross-sensor correspondences by extracting local geometric features, such as leveraging single-tree position consistency for heterogeneous airborne and terrestrial LiDAR registration [12], or extending two-dimensional features to three-dimensional matching. However, their robustness is often limited in the presence of large viewpoint variation, severe density inconsistency, and heavy occlusion. The normal distributions transform (NDT) represents the target point cloud as local probability distributions and optimizes the pose in the distribution space, which provides certain robustness in large-scale scenes [13]. Nevertheless, NDT still exhibits limitations in describing local structural details and accurately aligning planar boundaries. In recent years, learning-based registration methods have shown strong feature representation ability in complex scenes, but they usually require large training datasets and considerable computational resources, which limits their practical use in industrial applications such as grain warehousing robots. Alternatively, context-descriptor-based global localization solutions have been explored to guide heterogeneous robots using co-view information [14].

In summary, although ICP, point-to-plane ICP, GICP, and NDT have achieved substantial progress in general point cloud registration, it remains difficult to simultaneously guarantee accuracy, robustness, and engineering applicability in heterogeneous multi-LiDAR systems, especially under low-overlap, sparse–dense mixed, and plane-dominated industrial conditions. Therefore, rather than redesigning the underlying GICP optimizer, this paper improves the registration workflow on top of standard GICP and introduces a point-to-plane metric based on local planar models together with cross-LiDAR planar consistency verification.

Rather than redesigning the underlying GICP optimizer, this paper improves the registration workflow on top of standard GICP and introduces a point-to-plane metric based on local planar models together with cross-LiDAR planar consistency verification. Rather than redesigning the fundamental probabilistic optimization engine, the contribution of this workflow lies in the systematic engineering coupling of deterministic spatial constraints with statistical density equalization. Heterogeneous multi-LiDAR systems inherently generate extreme point density disparities and severe non-overlapping regions, often trapping standard GICP in local minima. By mathematically isolating the exact overlapping sub-space prior to registration, this workflow significantly reduces the risk of falling into local minima caused by invalid correspondences, thereby improving the robustness of standard GICP in heterogeneous registration tasks.

### 1.3. Main Contributions

This paper investigates point cloud registration for a heterogeneous multi-LiDAR system on a grain warehousing robot. Considering that the scene contains abundant planar structures and that the blind-spot LiDARs generate sparse point clouds while the main LiDAR produces relatively dense observations, a registration and verification framework is developed based on standard GICP. The proposed framework improves the registration process by optimizing multi-LiDAR registration organization, overlap-region constraints, and planar consistency evaluation. Different from recent studies that focus on targetless global calibration, probabilistic joint registration, or automated calibration pipelines, the present work emphasizes workflow improvement and post-registration planar consistency evaluation for a grain warehousing robotic platform.

First, a “one main LiDAR plus two blind-spot LiDARs” sensing configuration is established for the grain warehousing robot, and the spatial coverage, density distribution, and local structure characteristics of multi-source point clouds are analyzed. Second, a star-topology registration workflow is designed for sparse–dense and sparse–sparse mixed registration scenarios. The workflow introduces overlap-region cropping and voxel downsampling to reduce redundant data and noise, thereby improving the engineering applicability of standard GICP in heterogeneous point cloud registration. Third, a point-to-plane metric based on local planar models and a cross-LiDAR planar consistency verification strategy are introduced to evaluate structural alignment quality beyond the conventional point-to-point fitness score.

The main contributions of this paper are as follows:

- A point cloud registration workflow is developed for heterogeneous multi-LiDAR systems mounted on a grain warehousing robot, including a star-topology organization strategy suitable for sparse–dense and sparse–sparse mixed registration.
- A GICP-based registration flow improvement method is proposed. Overlap-region cropping and voxel downsampling are used to reduce invalid matches and improve the applicability of standard GICP in low-overlap heterogeneous scenarios.
- A planar consistency evaluation and cross-LiDAR verification mechanism is introduced. The proposed point-to-plane metric and planar consistency analysis improve the interpretability and credibility of registration results in terms of geometric structure.

## 2. Materials and Methods

### 2.1. Robotic Platform

To satisfy the mobility, payload capacity, and multi-sensor integration requirements of grain intake operations, a tracked intelligent grain warehousing robot platform was constructed. Grain storage environments usually contain loose grain accumulation, uneven slopes, heavy dust, and limited operating space. Therefore, the locomotion platform must provide both good obstacle-crossing capability and sufficient load-bearing capacity for LiDARs, onboard computing units, and communication modules. A tracked chassis was selected because of its large ground contact area, low ground pressure, good traversability, and superior anti-slip performance. This structure can adapt to complex ground conditions during entry and exit operations while providing sufficient installation space and load capacity for the perception system.

**Figure 1:**
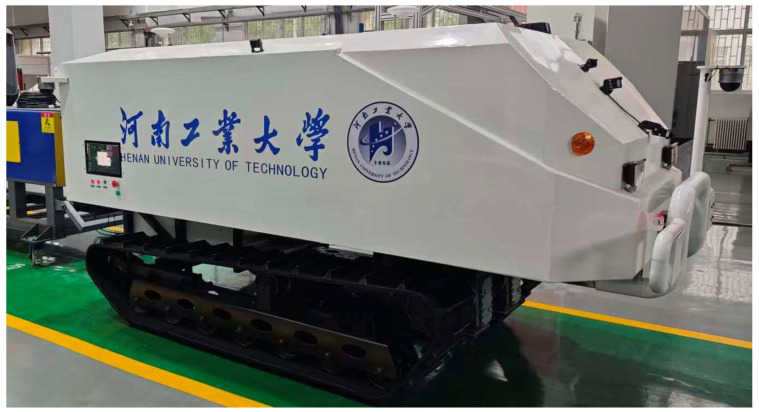
Photo of the intelligent grain warehousing robot experimental platform. (The Chinese characters on the robot body translate to ‘Henan University of Technology’).

The robot platform (Figure 1) consists of a tracked mobile chassis, a multi-LiDAR sensing unit, an onboard computing unit, and software modules for communication and coordinate management. The multi-LiDAR sensing unit is responsible for point cloud acquisition and coverage enhancement. The onboard computing unit performs point cloud preprocessing, registration, coordinate transformation, and result evaluation. The software system is responsible for multi-source data acquisition, coordinate unification, and inter-module communication. The platform provides the experimental basis for heterogeneous point cloud registration and multi-LiDAR fusion.

### 2.2. Heterogeneous Multi-LiDAR Configuration

To compensate for blind spots, severe near-field occlusion, and the difficulty of simultaneously satisfying far-field and near-field perception requirements, a heterogeneous “one main LiDAR plus two blind-spot LiDARs” configuration is adopted. The system consists of one high-line-count main LiDAR and two low-line-count auxiliary LiDARs, forming a multi-view and hierarchical three-dimensional sensing structure. The main LiDAR is mounted at the top center of the vehicle and mainly provides far-range scene perception, global scene mapping, and overall structural representation. The two auxiliary LiDARs are mounted around the vehicle body to compensate for near-field blind zones and local occluded regions. Through this cooperative configuration, near-field coverage is enhanced while preserving long-range structural perception.

The hardware configuration includes one 128-line RS-LiDAR-M1 as the main LiDAR and two 32-line RS-Bpearl units as blind-spot LiDARs. The main LiDAR generates dense point clouds with relatively complete planar structure representation, whereas the blind-spot LiDARs are sparse and more suitable for local coverage enhancement. Accordingly, the resulting multi-source point clouds exhibit clear heterogeneity in sampling density, local geometry, and viewpoint, which naturally forms sparse–dense and sparse–sparse mixed registration conditions.

**Figure 2:**
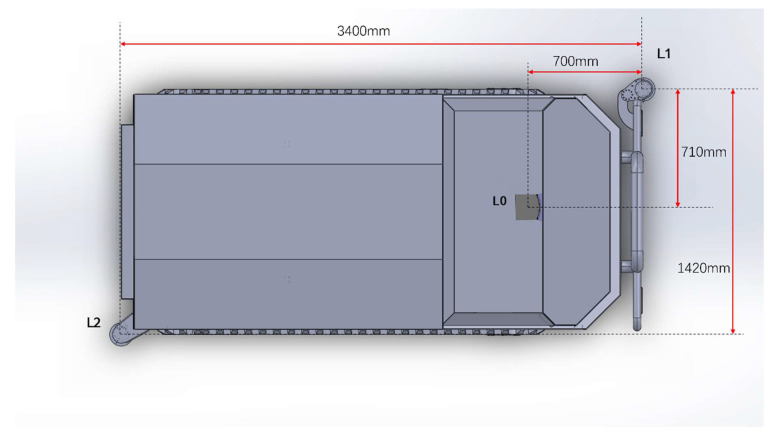
Dimensional drawing of LiDAR installation positions. L0 represents the main LiDAR, while L1 and L2 represent the two blind-spot LiDARs.

**Figure 3:**
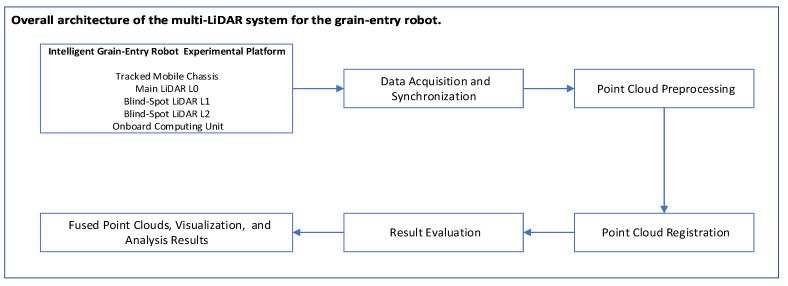
Overall architecture of the multi-LiDAR system for the grain warehousing robot.The arrows indicate the direction of data flow and processing sequence.

**Figure 4:**
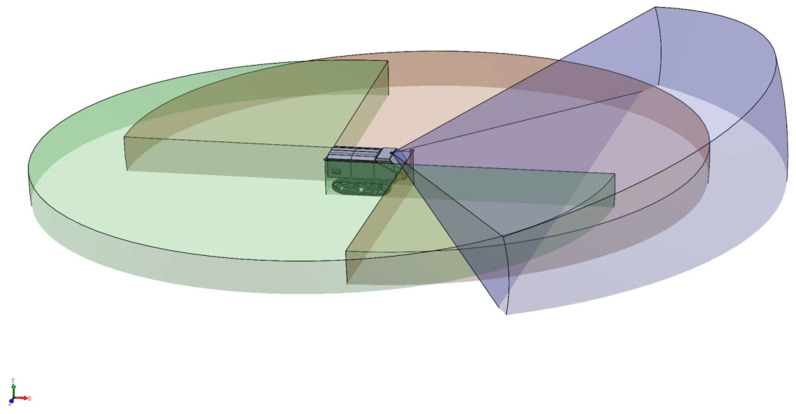
Field-of-view coverage of the three LiDAR sensors. The blue, brown, and green regions indicate the fields of view of the main LiDAR $L_{0}$, the blind-spot LiDAR ${\;L}_{1}$, and the blind-spot LiDAR $L_{2}$, respectively.

Specifically, the main LiDAR is installed at a height of 1.6 m, featuring a measurement range of 150 m, a horizontal field of view (FOV) of 120° (−60.0° to +60.0°), and a vertical FOV of 25° (−12.5° to +12.5°). The two blind-spot LiDARs are installed at a height of 1.4 m, with a measurement range of 30 m, a horizontal FOV of 0° to 360°, and a vertical FOV of 0° to 90°. The top-view coverage ranges are illustrated in Figure 2:

The spatial arrangement provides nearly 360° perception coverage around the vehicle on the horizontal plane and creates multi-level overlap in several regions. These overlap regions provide the geometric basis for cross-LiDAR registration, while differences in viewpoint and density make the registration process challenging.The overall architecture of the multi-LiDAR system and the three-dimensional field-of-view coverage are illustrated in Figure 3 and Figure 4, respectively.

### 2.3. Software Platform and Data Processing Pipeline

To realize synchronized acquisition, coordinate unification, and subsequent point cloud processing, the software system is implemented on Ubuntu 18.04 and ROS Melodic, together with PCL (version 3.3.4) and Eigen (version 3.3.4) for point cloud processing and matrix computation. This software environment supports real-time multi-LiDAR data access, preprocessing, coordinate transformation, and registration experiments. The node-based ROS architecture also facilitates modular implementation of sensing, preprocessing, registration, and visualization.

In the data processing pipeline, raw point clouds are first read by LiDAR driver nodes and published to a unified communication bus. The raw data are then filtered, cropped, and downsampled to reduce noise and redundancy. The preprocessed point clouds are subsequently sent to the registration module, where standard GICP is used to estimate the rigid transformations between LiDAR coordinate systems. After the transformations are obtained, the point clouds are transformed into a unified coordinate frame for fusion and visualization, and point-to-plane as well as cross-LiDAR planar consistency metrics are computed for quantitative analysis.

### 2.4. Coordinate Systems and Problem Formulation

To represent multi-source point clouds in a unified spatial reference, a world coordinate system W, a robot base coordinate system B, and LiDAR coordinate systems $L_{0}$, $L_{1}$, and $L_{2}$ are defined. The base coordinate system B is defined at the projection of the vehicle center onto the ground, while each LiDAR coordinate system is fixed to its own sensor body.

Mechanical measurement can provide an initial estimate of the extrinsic parameters between each LiDAR and the robot base frame. However, due to assembly errors and installation deviations, these initial extrinsics can only be used as coarse initial values. Therefore, the point cloud registration task in this paper is formulated as estimating more accurate rigid transformations between LiDAR coordinate systems under known coarse extrinsic initialization.

(1) $$T_{\mathrm{ri}}=\left[\begin{array}{cc} R_{\mathrm{ri}} & t_{\mathrm{ri}}\\ 0^{⊤} & 1 \end{array}\right]$$

where $R_{ri}$ ∈ SO(3) denotes the rotation matrix and $t_{ri}$ ∈ $R^{3}$ denotes the translation vector.

(2) $$p_{r}=R_{\mathrm{ri}}p_{i}+t_{\mathrm{ri}}$$

where $p_{i}$ is a point in LiDAR coordinate system $L_{i}$ and $p_{r}$ is the transformed point in the reference coordinate system.

In the registration process, $L_{1}$ is selected as the reference LiDAR frame, while the final fused point clouds are expressed in the vehicle coordinate system B.

### 2.5. GICP-Based Registration Flow Improvement

#### 2.5.1. Method Overview

The proposed method addresses low-overlap sparse–dense and sparse–sparse mixed registration in the heterogeneous multi-LiDAR system. Instead of directly applying registration to the full raw point clouds, the method improves the workflow before and after standard GICP by introducing overlap-region constraints, star-topology registration organization, and planar consistency evaluation.

**Figure 5:**
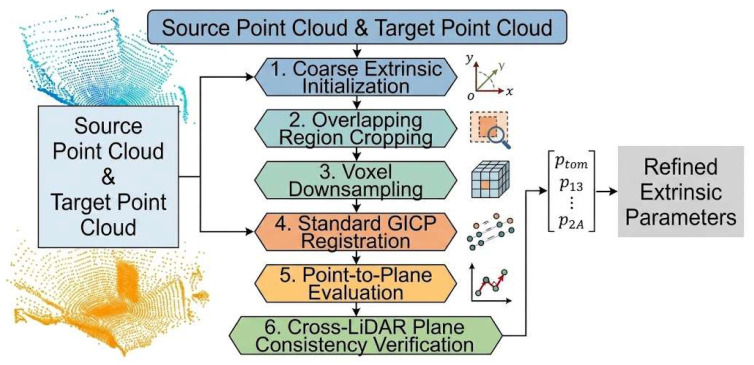
Workflow of the proposed GICP-based registration flow improvement method. The arrows indicate the processing sequence of the algorithm, and the different colors represent distinct functional stages.

The workflow, as illustrated in Figure 5, consists of the following steps:

- Coarse extrinsic initialization from mechanical measurement;
- Spatial cropping of source and target point clouds;
- Voxel downsampling to reduce point cloud size;
- Standard GICP-based rigid registration;
- Point-to-plane evaluation based on local planar models;
- Cross-LiDAR planar consistency verification on representative planar regions.

#### 2.5.2. Overlap-Region-Constrained Preprocessing

Low-overlap heterogeneous point clouds often contain many points unrelated to valid matching regions. Directly registering the full point clouds increases the risk of invalid correspondences and decreases optimization stability. Therefore, spatial cropping is first applied according to sensor installation positions and field-of-view relations, and only the points within the potential overlap region are retained [15].

(3) $$P_{c}=p_{i}=(x_{i},y_{i},z_{i}{)}^{T}\in P|x_{min}\leq x_{i}\leq x_{max},y_{min}\leq y_{i}\leq y_{max},z_{min}\leq z_{i}\leq z_{max}|$$

This operation concentrates the registration constraints on geometrically relevant regions and reduces the influence of distant noise and unrelated structures.

After spatial cropping, the main LiDAR point cloud remains relatively dense. To reduce computational cost and alleviate the influence of density imbalance, voxel-grid filtering is used for downsampling. Let $V_{j}$ = {$p_{j,1},p_{j,2},\ldots ,p_{j,Nj}$} denote the point set in the j-th voxel. The centroid of this voxel is computed as

(4) $${\overset{-}{p}}_{j}=\frac{1}{N_{j}}\sum_{k=1}^{N_{j}}p_{k}$$

The downsampled point cloud is then represented as

(5) $$P_{d}=\{{\overset{-}{p}}_{j}{\}}_{j=1}^{M}$$

where M is the number of retained voxels.

#### 2.5.3. Standard GICP Registration

GICP introduces local covariance modeling into the ICP framework to unify point-to-point and point-to-plane constraints. For the local neighborhood N(q) of a point q, the covariance matrix is given by

(6) $$C=\frac{1}{K}\sum_{k=1}^{K}{\left(q_{k}-\overset{-}{q})(q_{k}-\overset{-}{q}\right)}^{T}$$

where $\overset{-}{q}$ is the neighborhood mean and K is the number of neighboring points.

For a correspondence pair ($p_{i}^{s}$, $p_{i}^{t}$), the residual is defined as

(7) $$r_{i}=p_{i}^{t}-(Rp_{i}^{s}+t)$$

The GICP objective function can be written as

(8) $$\underset{R,t}{\min}\sum_{i=1}^{N}r_{i}^{T}(C_{i}^{t}+RC_{i}^{s}R^{T}{)}^{-1}r_{i}$$

where N is the total number of matched correspondence pairs, and $C_{i}^{s}$ and $C_{i}^{t}$ represent the local covariance matrices of the source point $p_{i}^{s}$ and the target point $p_{i}^{t}$, respectively. This objective function is iteratively optimized in a weighted least-squares manner. In this study, standard PCL GICP is adopted as the core registration engine.

#### 2.5.4. Star-Topology Multi-LiDAR Registration Organization

The choice of the reference coordinate frame directly affects the stability of multi-LiDAR registration. The reference LiDAR should not be selected merely according to point density, but rather according to the effective overlap with the other sensors.

**Figure 6:**
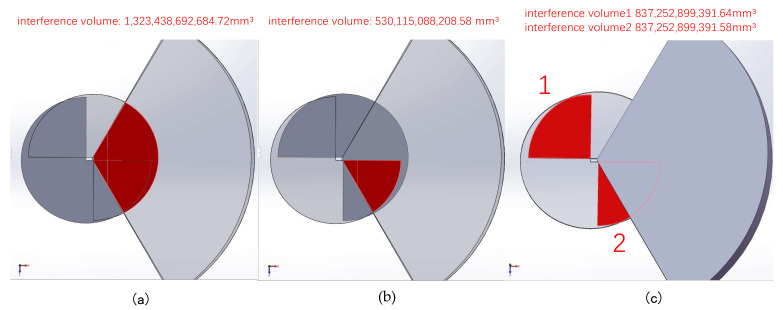
Schematic diagram of the field-of-view overlap volumes for the multi-LiDAR system. (**a**) overlap volume between the main LiDAR $L_{0}$ and the blind-spot LiDAR $L_{1}$; (**b**) overlap volume between the main LiDAR $L_{0}$ and the blind-spot LiDAR $L_{2}$; (**c**) overlap volumes between the two blind-spot LiDARs $L_{1}$ and $L_{2}$. The red regions indicate the specific intersection (interference) volumes.

The overlap areas correspond to the geometric intersection regions of the respective sensor fields of view, as illustrated in Figure 6. Based on a quantitative 3D interference analysis conducted in SolidWorks (version 2021), the volume of the overlapping interference region between the main LiDAR $L_{0}$ and the blind-spot LiDAR $L_{1}$ is 1323.4387 $m^{3}$. The overlap volume between $L_{0}$ and the other blind-spot LiDAR $L_{2}$ is 530.1151 $m^{3}$, while the overlap volume between $L_{1}$ and $L_{2}$ reaches 1674.5058 $m^{3}$. Since the blind-spot LiDAR $L_{1}$ exhibits significantly larger overlapping volumes with both $L_{0}$ and $L_{2}$ compared to alternative pairs, it provides the strongest spatial constraints for the entire system. Therefore, $L_{1}$ is selected as the central reference LiDAR frame for the subsequent star-topology registration workflow.

In the present platform, the blind-spot LiDAR $L_{1}$ has relatively larger overlap with both the main LiDAR $L_{0}$ and the other blind-spot LiDAR $L_{2}$. Therefore, $L_{1}$ is selected as the reference LiDAR frame during the registration process.

Based on this consideration, a star-topology registration organization is adopted. Rigid transformations $T_{10}$ and $T_{12}$ are first estimated for $L_{0}$→$L_{1}$ and $L_{2}$→$L_{1}$, respectively. After registration in the $L_{1}$ frame, all point clouds are further transformed into the vehicle coordinate system B through the known extrinsic transformation TB1 from $L_{1}$ to B.

(9) $$p_{B}=T_{B1}T_{10}p_{0}$$

(10) $$p_{B}=T_{B1}p_{1}$$

(11) $$p_{B}=T_{B1}T_{12}p_{2}$$

**Figure 7:**
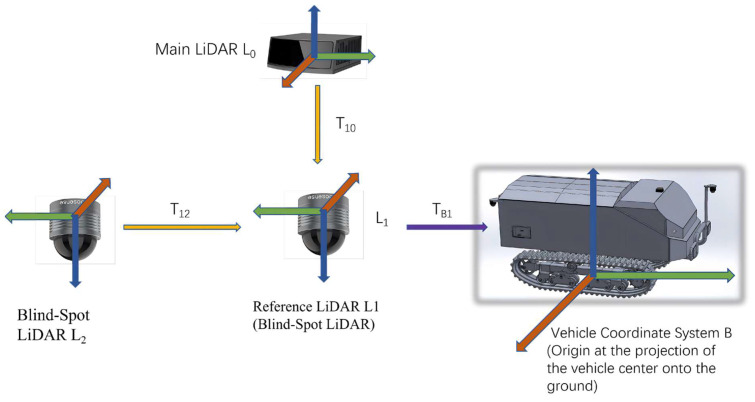
Star-topology multi-LiDAR registration organization with $L_{1}$ as the reference LiDAR and B as the final unified coordinate system.The yellow and purple arrows indicate the direction of rigid transformations (yellow for cross-LiDAR registration and purple for the final transformation to the vehicle base frame), while the red, green, and blue arrows represent the 3D coordinate systems.

Thus, as illustrated in Figure 7, $L_{1}$ serves as the registration reference node, while B is used as the final unified output frame for point cloud fusion. Compared to a sequential daisy-chain registration (e.g., $L_{0}\to L_{1}\to L_{2}$), where the transformation error from the first pair is inherently propagated and accumulated into subsequent pairs, the star-topology directly aligns each auxiliary LiDAR to the central reference LiDAR independently. This configuration completely decouples the registration processes, preventing progressive error accumulation and ensuring that each pairwise alignment benefits from the maximum possible overlapping region.

#### 2.5.5. Point-to-Plane Evaluation Based on Local Planar Models

The fitness score of standard GICP is mainly based on average point-to-point residuals. In environments dominated by planar structures such as walls, floors, and equipment surfaces, this metric cannot fully reflect structural alignment quality. Therefore, a local planar consistency evaluation is introduced after registration.

Specifically, for each registered source point $p_{i}$, we identify its nearest neighbor $q_{i}$ in the target point cloud. A local planar model $\pi_{i}$ is then fitted from the neighborhood of $q_{i}$ and represented as:

(12) $$a_{i}x+b_{i}y+c_{i}z+d_{i}=0$$

The point-to-plane distance of a registered source point p to the local plane π is computed as

(13) $$d_{\pi }\left(p_{i},\pi_{i}\right)=\frac{\left|a_{i}x_{p_{i}}+b_{i}y_{p_{i}}+c_{i}z_{p_{i}}+d_{i}\right|}{\sqrt{a_{i}^{2}+b_{i}^{2}+c_{i}^{2}}}$$

**Figure 8:**
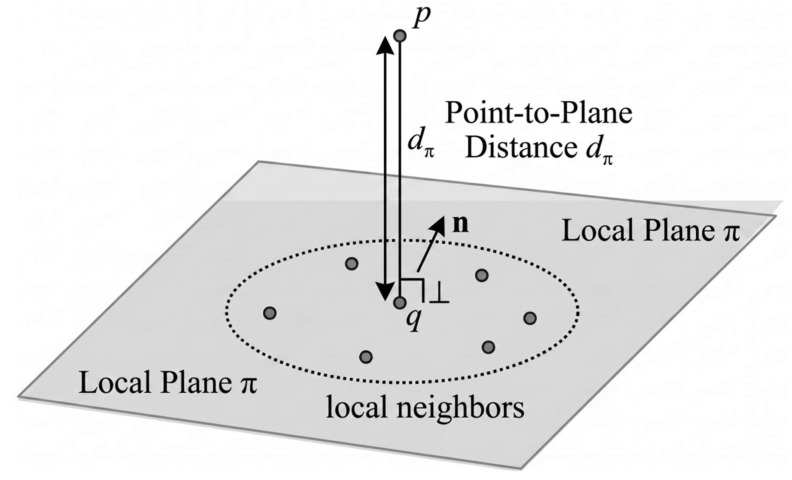
Point-to-plane evaluation based on a local planar model. The small grey circles represent the local neighbors used to fit the plane π, the arrow n denotes the normal vector of the local plane, and $d_{\pi }$ indicates the perpendicular point-to-plane distance from point p.

The overall point-to-plane evaluation score is then defined as

(14) $$E_{\mathrm{plane}}=\frac{1}{N}\sum_{i=1}^{N}d_{\pi }(p_{i},\pi_{i})$$

where *N* is the number of valid correspondences within the distance threshold. A smaller value indicates better structural alignment quality. This metric is used for post-registration evaluation rather than being directly embedded into the GICP optimization process, and the corresponding geometric relationship is illustrated in Figure 8.

#### 2.5.6. Cross-LiDAR Planar Consistency Verification

To further verify the geometric consistency of the multi-LiDAR system, several representative planar regions are manually selected from the fused point cloud, and the corresponding planes are fitted from different LiDAR point clouds. The normal-angle error between two fitted planes is defined as

(15) $$\theta_{\mathrm{ij}}=\;\arccos\left(n_{i}^{T}n_{j}\right)$$

A smaller $\theta_{ij}$ indicates higher directional consistency. Together with the point-to-plane distance in the same region, this metric evaluates both directional and positional consistency of the registered point clouds.

## 3. Results

### 3.1. Experimental Setup

The experiments were conducted on the tracked grain warehousing robot equipped with one 128-line main LiDAR and two 32-line blind-spot LiDARs. The software platform was based on Ubuntu 18.04, ROS Melodic, PCL, and Eigen. During the experiments, the robot remained stationary while multi-LiDAR point clouds were collected from different viewing directions. The experiments were carried out in the innovation laboratory building of the College of Mechanical and Electrical Engineering, Henan University of Technology. The scene contained large planar structures and mechanical equipment, approximating an empty storage environment.

Regarding the detailed implementation and reproducibility, all algorithms were executed on an onboard Y_Engion embedded edge computing device, which is equipped with an NVIDIA Jetson AGX Xavier processor and 32 GB of RAM. The core point cloud processing relied on PCL version 1.10. To ensure temporal consistency, the LiDAR sensors were synchronized via hardware PTP and ROS message filters. For the standard GICP algorithm applied in our workflow, the specific parameter settings were strictly controlled: the voxel grid leaf size for downsampling was set to 0.1 m, the maximum correspondence distance was 1.5 m, and the maximum number of iterations was set to 50. The transformation epsilon (convergence criterion) was set to $1\;\times \;10^{-10}$, and the neighbor search size for covariance estimation was set to k = 30.

To ensure consistency, all datasets were processed according to the same workflow. Raw point clouds from the three LiDARs were first read and initialized by coarse extrinsics from mechanical measurement. The point clouds were then cropped and downsampled, and two registration tasks were performed. Finally, the results were transformed into the vehicle coordinate system B and evaluated using point-to-plane errors and cross-LiDAR planar consistency metrics.

### 3.2. Comparative Methods and Evaluation Metrics

ICP, point-to-plane ICP, NDT, and the proposed method were selected as comparative baselines. All methods used the same coarse initial extrinsics, spatial cropping region, voxel-grid size, and maximum correspondence distance to ensure a fair comparison, as listed in Table 1. The advantage of the proposed method does not lie in changing the underlying data, but in improving the registration workflow through overlap-region constraints, star-topology organization, and planar consistency evaluation.

Registration performance was evaluated from both geometric accuracy and computational efficiency. Geometric accuracy was mainly measured by the average point-to-plane error, which describes the fitting quality of registered source points to local planar models in the target point cloud. In addition, planar normal-angle errors and point-to-plane distances were computed in representative planar regions to verify structural geometric consistency. Running time was used to characterize computational efficiency.

### 3.3. Quantitative Results

Table 2 summarizes the quantitative comparison for the two heterogeneous point cloud registration tasks. In the $L_{0}-L_{1}$ sparse–dense registration task, the proposed method achieved a point-to-plane error of 0.1487 m, which is substantially lower than those of ICP (1.4125 m), point-to-plane ICP (0.3463 m), and NDT (0.3658 m). The corresponding running times were 53.781 ms, 229.019 ms, 98.322 ms, and 54.480 ms, respectively.

In the $L_{1}-L_{2}$ sparse–sparse registration task, the proposed method achieved a point-to-plane error of 0.1090 m, again outperforming ICP (1.5473 m), point-to-plane ICP (0.3968 m), and NDT (0.2824 m). The corresponding running times were 7.702 ms, 27.037 ms, 15.000 ms, and 7.166 ms, respectively. These results indicate that the proposed method yields lower structural alignment errors in both tasks while maintaining computational performance on the same order as NDT.

**Figure 9:**
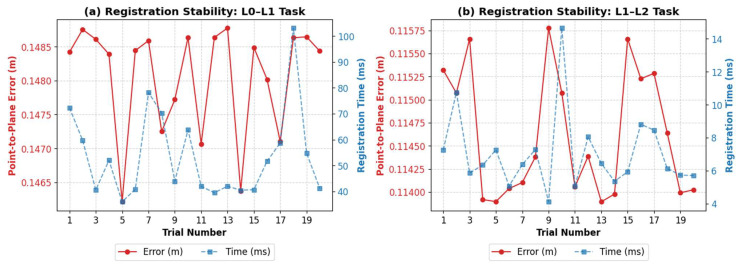
Performance stability of the proposed registration workflow across 20 independent Monte Carlo perturbation trials. The solid red lines with circular markers indicate the point-to-plane error (**left axis**), while the dashed blue lines with square markers represent the registration time (**right axis**).

Furthermore, the statistical results from the 20-trial Monte Carlo perturbation tests highlight the exceptional stability of the proposed method. For each trial, randomized translational perturbations drawn from a uniform distribution within [−0.2 m, +0.2 m] and rotational perturbations uniformly sampled within [−5°, +5°] were injected into the initial coarse extrinsic parameters. Crucially, all compared baseline methods were initialized with the exact same randomized perturbations. The detailed iteration-by-iteration execution logs and convergence values for all 20 independent trials are archived in File S1 (Monte_Carlo_Log_L1_L2.txt). Additionally, sample point cloud data, parameter profiles, and replication guides are available in Data S1 (1.pcd, 2.pcd), File S2 (parameters.yaml), and File S3 (README.md), respectively. In the $L_{0}-L_{1}$ sparse–dense registration task, the proposed method maintained an average point-to-plane error of 0.1481 ± 0.0008 m with an average registration time of 53.57 ± 17.12 ms. In the $L_{1}-L_{2}$ sparse–sparse registration task, the average point-to-plane error was 0.1146 ± 0.0007 m with an average time of 7.03 ± 2.29 ms. Figure 9 illustrates the distribution of registration scores and times across the 20 trials. The extremely low standard deviations (below 0.001 m in both tasks) confirm that the proposed algorithm can reliably converge to high-quality structural alignments despite significant initial pose uncertainties.

### 3.4. Qualitative Results and Planar Consistency Analysis

**Figure 10:**
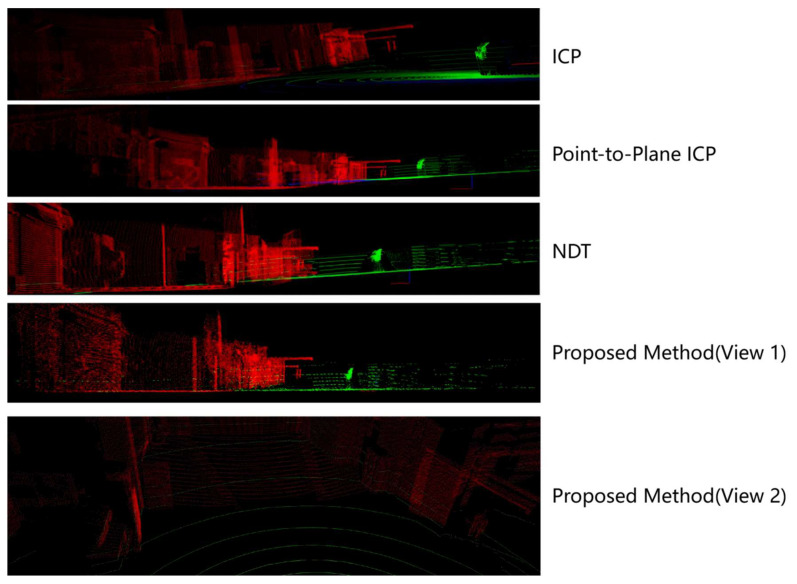
Registration visualization of $L_{0}-L_{1}.$ The red and green points represent the point clouds from the main LiDAR $L_{0}$ and the reference LiDAR $L_{1}$, respectively.

**Figure 11:**
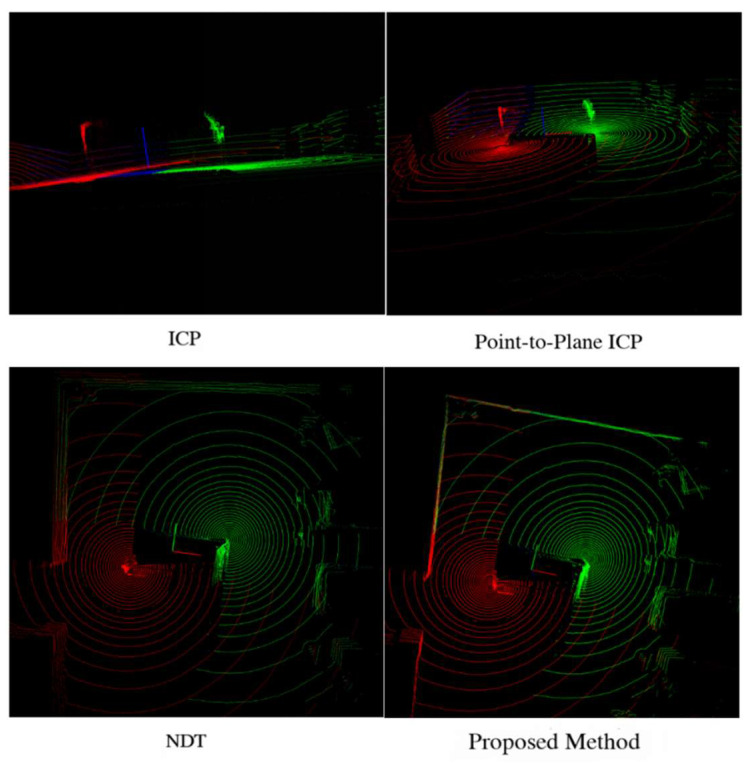
Registration visualization of $L_{1}-L_{2}.$ The red and green points represent the point clouds from the reference LiDAR $L_{2}$ and the blind-spot LiDAR $L_{1}$, respectively.

The qualitative registration results for the $L_{0}-L_{1}$ and $L_{1}-L_{2}$ tasks are visually illustrated in Figure 10 and Figure 11, respectively. The results show that ICP suffers from obvious misalignment and boundary drift in large planar regions such as walls and floors. Point-to-plane ICP improves the results to some extent but still exhibits local offsets in the heterogeneous low-overlap scenario. NDT produces relatively stable global alignment, but local distortion or ghosting remains near planar intersections and equipment boundaries. In contrast, the proposed method achieves more continuous alignment with smaller residual errors on representative structural planes such as walls, floors, and equipment surfaces.

To further analyze structural geometric consistency, six representative planar regions were selected from the fused point cloud map. To ensure a comprehensive evaluation, these regions were chosen to cover typical orthogonal structural surfaces inherent to the grain warehouse environment, specifically including two large wall segments, two floor segments, and two metallic equipment surfaces. For the $L_{0}\to L_{1}$ group, the normal-angle errors were 0.81835°, 0.28069°, and 0.26874°, and the corresponding point-to-plane distances were 0.0054 m, 0.1224 m, and 0.0208 m. For the $L_{2}\to L_{1}$ group, the normal-angle errors were 0.58990°, 0.79804°, and 0.08103°, and the corresponding point-to-plane distances were 0.0556 m, 0.1004 m, and 0.0331 m. Overall, all planar normal-angle errors were below 1°, and all point-to-plane distances remained below 0.13 m, indicating good consistency in both planar orientation and position, as summarized in Table 3.

## 4. Discussion

The results demonstrate that the proposed method provides a favorable balance between accuracy and efficiency in low-overlap heterogeneous point cloud registration. The main advantages lie in two aspects. First, overlap-region cropping and downsampling reduce the influence of invalid correspondences on standard GICP and improve registration stability [16]. Second, the point-to-plane metric based on local planar models extends the analysis from conventional point-to-point residuals to structural alignment quality, which is more suitable for plane-dominated grain-storage environments.

Furthermore, it is essential to contextualize the achieved registration accuracy within the actual operational requirements of the grain warehousing robot. In large-scale grain storage scenarios, global 3D mapping frameworks typically adopt a voxel resolution of 0.15 m to 0.20 m to balance memory consumption and representation detail. The average point-to-plane errors achieved by our method (0.1487 m and 0.1090 m) are strictly bounded by this standard voxel size, effectively avoiding the “ghosting” effect in the fused map. Moreover, for autonomous locomotion, the robot’s local path planner typically applies an obstacle inflation radius of >0.3 m. The structural alignment deviation is well within this safety margin, ensuring that cross-sensor spatial noise will not trigger false collision alarms.

It should also be noted that the current experiments were conducted in an approximately empty storage environment, and the robot remained stationary while point clouds were collected by changing the viewing direction. Therefore, the present results mainly verify the effectiveness of the proposed workflow under static multi-LiDAR registration conditions. Further studies are still required for online registration during dynamic grain-entry operations, severe occlusion conditions, and more complex real grain-storage environments.

## 5. Conclusions

This paper addressed low-overlap point cloud registration in a heterogeneous multi-LiDAR system for a grain warehousing robot and proposed a registration workflow improvement method based on standard GICP together with planar consistency evaluation. By combining overlap-region cropping, voxel downsampling, and star-topology registration organization, the proposed method improved the stability of heterogeneous point cloud registration. In addition, a point-to-plane metric based on local planar models and a cross-LiDAR planar consistency verification strategy were introduced to better evaluate structural alignment quality.

Experimental results showed that the proposed method outperformed ICP, point-to-plane ICP, and NDT in both sparse–dense and sparse–sparse registration tasks while maintaining favorable computational efficiency. The qualitative results and planar consistency analysis further confirmed the effectiveness of the method on typical planar structures such as walls, floors, and equipment surfaces. Overall, the proposed workflow provides a reliable geometric basis for multi-sensor perception, environment mapping, and autonomous operation of grain warehousing robots. It should be explicitly noted that the current experimental validation remains limited to a stationary robotic platform within a structured storage-like environment. Therefore, the implications for autonomous operation discussed in this study are strictly bounded to static or semi-static registration scenarios.

## Figures and Tables

**Table 1:** Registration parameter settings.

Parameter (Symbol/Description)	Value
Cropping range (*X*-axis) ($x_{\min}$, $x_{\max}$)	$L_{0}$$–L_{1}$ (4.0 m, 20.0 m), $L_{1}$$–L_{2}$ (−20.0 m, −0.5 m)
Cropping range (*Y*-axis) ($y_{\min}$, $y_{\max}$)	$L_{0}$$–L_{1}$ (−20.0 m, 20.0 m), $L_{1}$$–L_{2}$ (0.0 m, 20.0 m)
Cropping range (*Z*-axis) ($z_{\min}$, $z_{\max}$)	$L_{0}$$–L_{1}$ (−1.0 m, 5.0 m), $L_{1}$$–L_{2}$ (−1.0 m, 5.0 m)
Voxel size (leaf size)	0.1 m
Maximum correspondence distance	1.5 m
Maximum iterations	50
Transformation epsilon ($\epsilon_{trans}$)	1 × 10^−10^
Euclidean fitness epsilon ($\epsilon_{fit}$)	0.01
Neighborhood size for local plane fitting (k)	30
Reciprocal correspondences	Yes

**Table 2:** Quantitative comparison of different registration methods.

	$L_{0}$ $–L_{1}$		$L_{1}$ $–L_{2}$	
Method	Point-to-Plane Error (m)	Time (ms)	Point-to-Plane Error (m)	Time (ms)
ICP	1.4125	229.019	1.5473	27.037
Point-to-Plane ICP	0.3463	98.322	0.3968	15.000
NDT	0.3658	54.480	0.2824	7.166
Proposed Method	0.1487	53.781	0.1090	7.702

**Table 3:** Cross-LiDAR planar consistency statistics.

Registration Pair	Plane	Normal-Angle Error (°)	Average Point-to-Plane Distance (m)
$L_{0}\to L_{1}$	Plane 1 (Wall)	0.81835	0.0054
	Plane 2 (Floor)	0.28069	0.1224
	Plane 3 (Equipment)	0.26874	0.0208
$L_{2}\to L_{1}$	Plane 4 (Wall)	0.58990	0.0556
	Plane 5 (Floor)	0.79804	0.1004
	Plane 6 (Equipment)	0.08103	0.0331

## Data Availability

Data is contained within the Supplementary Materials. A representative point cloud data sample and the configuration parameter file used in this study have been provided as Supplementary Files to support reproducibility.

## Supplementary Materials

The following supporting information can be downloaded at: https://www.mdpi.com/article/10.3390/s26113447/s1, File S1: Monte_Carlo_Log_L1_L2.txt (Full execution logs and statistical details of the 20-trial Monte Carlo perturbation tests); File S2: parameters.yaml (Algorithmic configuration and parameter settings); File S3: README.md (Instructions and guide for experimental replication); Data S1: 1.pcd and 2.pcd (Sample multi-source heterogeneous point cloud datasets used for registration evaluation).

## Abbreviations

LiDAR	Light Detection and Ranging
GICP	Generalized Iterative Closest Point
ICP	Iterative Closest Point
NDT	Normal Distributions Transform
FOV	Field of View
ROS	Robot Operating System
PCL	Point Cloud Library
